# Mortality, Causes of Death, and Predictors of Death among Patients On and Off Opioid Agonist Treatment: Results from a 19-Year Cohort Study

**DOI:** 10.1159/000525694

**Published:** 2022-08-23

**Authors:** Ivar Skeie, Thomas Clausen, Arne Jan Hjemsæter, Anne Signe Landheim, Bent Monsbakken, Magne Thoresen, Helge Waal

**Affiliations:** ^a^National Advisory Unit on Concurrent Substance Abuse and Mental Health Disorders, Department of Mental Health, Innlandet Hospital Trust, Ottestad, Norway; ^b^Norwegian Centre for Addiction Research, Institute of Clinical Medicine, University of Oslo, Oslo, Norway; ^c^Faculty of Social and Health Sciences, Inland Norway University of Applied Sciences, Elverum, Norway; ^d^Department of Mental Health, Innlandet Hospital Trust, Reinsvoll, Norway; ^e^Department of Biostatistics, Institute of Basic Medical Sciences, University of Oslo, Oslo, Norway; ^f^National Advisory Unit on Substance Use Disorder Treatment, Oslo University Hospital, Oslo, Norway

**Keywords:** Opioid use disorder, Opioid agonist treatment, Mortality, Causes of death, Retention in treatment, Ageing

## Abstract

**Background:**

Mortality is increased among people with opioid use disorder but reduced while on opioid agonist treatment (OAT). However, the impact of patient and treatment characteristics on mortality and causes of death is insufficiently studied.

**Objectives:**

The objective of this study was to explore mortality and causes of death and examine the impact of patient and treatment characteristics on mortality in an OAT cohort with high retention in treatment.

**Methods:**

*Design*: longitudinal cohort study. *Setting*: Norway. *Observation period*: time from OAT start as of 1998 until death or end of 2016, 2,508 person-years (PY) in total. *Sample*: 200 persons starting OAT 1998–2007. *Data sources*: hospital records, interviews, the Norwegian Cause of Death Registry, Statistics Norway.

**Results:**

Retention: 86.4% of the observation period was on OAT, 9.0% off, 4.6% unknown OAT status. All-cause crude mortality rate per 100 PY during the whole observation period was 1.64 (95% CI: 1.19–2.20), for deaths of somatic cause 0.88 (0.56–1.31), for drug-induced deaths 0.44 (0.23–0.76), and traumatic deaths 0.24 (0.10–0.50). Off-versus-on-OAT all-cause mortality ratio was 2.31 (1.00–4.85). On OAT, 58% of the deaths were of somatic cause and 21% drug-induced; off OAT, 38% of somatic cause and 50% drug-induced. Increasing baseline age and rate of somatic hospital treatment episodes were independently associated with *increased* all-cause mortality risk, while increasing rate of in-patient psychiatric treatment episodes was associated with *reduced* risk. Increasing duration of nicotine and cannabis use and alcohol dependence as well as increasing severity of polydrug use were associated with increased all-cause and somatic mortality adjusted for age and sex.

**Conclusion:**

The long observation period made it possible to demonstrate the importance of long-term retention in OAT to reduce mortality. Further, the preponderance of somatic and reduction of drug-induced causes of death during OAT underlines the need for follow-up of chronic diseases and health-promoting lifestyle changes. These findings add to the knowledge about long-term OAT effects, not least in ageing OAT populations.

## Introduction

All-cause mortality and especially mortality due to overdose and diseases directly related to high-risk drug-use behaviours like injecting is increased among people with opioid use disorder. Traumatic deaths and deaths from acute and chronic somatic diseases also occur more frequently. A 2020 systematic review and meta-analysis presents an all-cause crude mortality rate (CMR) of 1.59 deaths per 100 person-years (PY) (95% confidence interval [CI] 1.40–1.80) and a standardized mortality ratio (SMR) of 10.03 (7.64–13.17) among people using non-medical opioids [1], but this varies by setting.

Opioid agonist treatment (OAT) is the most used and widespread pharmacotherapy for opioid use disorder. It is well-established that all-cause CMR and SMR are reduced on compared to off OAT largely due to reduced overdose mortality [2, 3, 4]. A recent systematic review also documents reduced suicide, cancer, alcohol-related, and cardiovascular mortality risk on OAT [5]. However, the impact of risk factors and treatment characteristics on mortality is insufficiently studied, and many studies are characterized by relatively short treatment and follow-up time.

Here, we analyse these relationships in a cohort study that comprises the period from introduction of OAT as a national treatment programme in Norway in 1998 until 2016. The availability of longitudinal data from specialist psychiatric, somatic, and addiction treatment services as well as mortality and population data from national statistics enabled us to study mortality on and off OAT and associations between mortality and key patient and treatment characteristics. Therefore, the long follow-up time and high follow-up rate, the high age of the cohort [6], and high retention in OAT in Norway [7] make the study relevant, despite the limited cohort size.

The aims of the study were (a) to estimate CMRs for the whole observation period as well as on and off OAT, (b) to estimate all-cause SMR, (c) to explore causes of death, and (d) to examine the impact of patient and treatment characteristics on mortality and whether these differed between persons who died of somatic versus other causes.

## Materials and Methods

### Design

The study has a longitudinal cohort design in which the date of the participants' first OAT start are defined as their study entry date.

### Setting

In Norway, OAT is organized within the secondary level health care, and specialist OAT teams assess referrals, start and supervise treatment, and cooperate closely with general practitioners and municipal health and social services [8]. The national programme was started in 1998 and has evolved from an originally high-threshold rehabilitation-oriented profile [9] to combined rehabilitation and low-threshold harm-reduction goals [10]. Retention in treatment has been high from the start [7]. The Norwegian OAT population is among the oldest in Europe [6]. Reduced mortality on OAT compared with periods before and after is previously documented [10, 11, 12]. During 2014–2015, 45% of deaths on treatment were due to somatic diseases, 42% were drug-induced, and 12% were due to traumatic causes [13].

### Sample

**Fig. 1:**
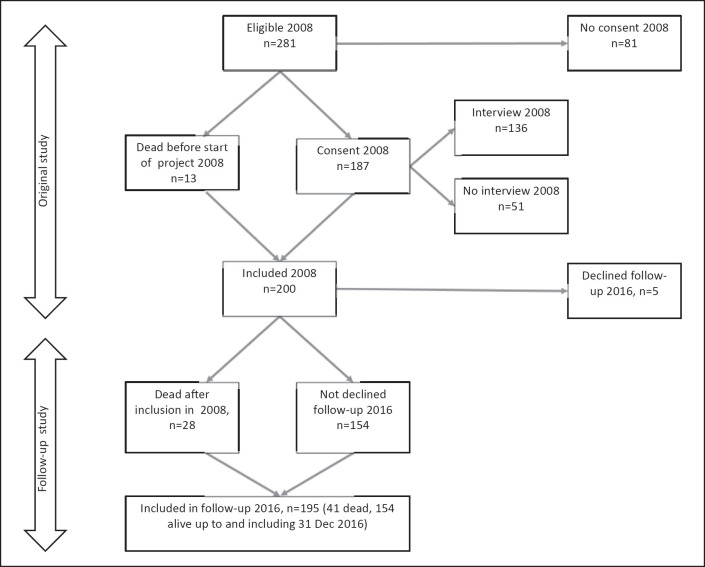
Flowchart: cohort established 2007/2008, participants starting OAT between 1 January 1998 and 30 June 2007. Thirteen persons who died after first start of OAT but before project start in 2007/2008 were included. The follow-up cohort consisted of 195 persons on 31 December 2016, of whom 41 were dead.

The cohort was established in 2007–2008 and recruited from the county Innlandet (Fig. 1). The recruitment procedure is previously described in detail [14]. The eligibility criterion was first OAT start between 1 January 1998 and 30 June 2007. Out of 281 eligible, 13 who died before the cohort was established were included and 187 consented to participate, rendering a cohort of 200 participants in 2008. Five participants declined to participate in the 2016 follow-up and another 28 died after study inclusion; thus, the follow-up cohort comprised 195 participants including 41 deceased (concerning inclusion of the deceased, see Statement of Ethics). Key demographic, substance use, mental problem, and OAT characteristics did not differ largely from the national and catchment-area OAT populations in 2007 and as described in the national 2007 OAT status report [15].

### Observation Period

The individual observation period was from the first OAT start until death or the end of the study on 31 December 2016. OAT start date was set to the 15th of the month of the first OAT inclusion. Time on OAT was defined as time on agonist medication including the first 5 days after the last reported medication intake. Time off OAT was defined as time out of OAT after the first inclusion in one or consecutive periods. Time with no information about OAT status (on or off OAT) was defined as time of unknown OAT status. Thus, the total observation time was time on and off OAT and time with unknown OAT status. OAT status was based on record information from the specialist OAT teams.

### Data Sources and Collection

The first study on this cohort dates back to 2008–2009 [14, 16]. The data in this follow-up study include the following data from the first study: (a) records from the specialist OAT teams 1998–2008/2009, (b) somatic hospital records as of 5 years before the first OAT entry until 2008–2009, (c) information from structured interviews 2007–2008 (mainly face-to-face, a few per telephone) with 136 out of 187 alive participants about personal data and with retrospective information about substance use history as well as education and employment history. In the follow-up study, record data from the specialist OAT teams 2008/2009–2016, and (d) secondary level in- and outpatient psychiatric and substance use disorder (SUD) treatment as of 2000 through 2016 for all participants were added. Further, (e) mortality data as of 1998 through 2016 including causes of death for the whole cohort was collected from the Norwegian Cause of Death Registry. To calculate SMRs, we used (f) Norwegian population statistics from the reference year 2008 (mid-year of the observation period) [17, 18].

### Measures and Covariates

All-cause and diagnose group-specific CMRs and all-cause SMRs for the total observation period were estimated for the original 200 participants (2,508 PY). All-cause off-versus-on-OAT CMR ratios were based on follow-up data from 195 participants, and time with unknown OAT status was excluded. Causes of death were divided into three main categories with subgroups based on principle cause of death: somatic disease (cardiovascular, cancer − not liver, liver − including liver cancer, infectious diseases, and respiratory disorders), drug-induced deaths (following the ICD-10 definitions used by the European Monitoring Centre for Drugs and Drug Addiction) [19, 20], traumatic deaths (suicide, unintentional accidents, homicide), and deaths of unknown cause.

Key patient and treatment characteristics that might be associated with mortality risk based on literature and theoretical considerations were chosen as covariates in the regression analysis. Covariates in the multiple regression models were baseline age (defined as age 1 January 1998, i.e., before anyone had started OAT), sex [2, 3, 4, 5], early (1998–2003) versus late (2004–2007) first start of OAT [10] as well as rate of hospital treatment episodes for somatic diseases before and during OAT until 2009 available from the original study (later data not available) [14, 16, 21] and rate of in-patient psychiatric treatment episodes (SUD treatment not included) 2000–2016 [22, 23, 24], and drug-use-history data. Duration data on alcohol dependence and drug and nicotine use were based on the interviews conducted in 2007–2008. Severity of polydrug use was registered as the number out of six specified substance groups that the participant had either been dependent on (alcohol) or used in a non-medical way (benzodiazepines/z-hypnotics, amphetamines, cocaine, cannabis, and opioids) for more than 5 years, rendering a number on a scale from 0 to 6.

### Statistics

Sample characteristics by dead/alive at end of study were explored by descriptive statistics, and differences between groups were analysed by χ^2^ test for categorical variables and *t* test (normally distributed) or Mann Whitney U test (non-normally distributed) for continuous variables, using SPSS v. 26 (IBM Corporation, Armonk, NY, USA). Estimations of CMRs and SMRs were performed in the OpenEpi calculator [25]. CIs for CMRs and SMRs and *p* value for off-versus-on-OAT CMR ratio were estimated using the mid-*P* exact test [26]. Associations between covariates and mortality risk were estimated as hazard ratios in Cox regression models with time since first start of OAT as time scale. Key covariates showing statistically significant (or close to significant) hazard ratios in bivariate analysis were included in the multiple models. In the primary analysis, we estimated three different regression models; one for death of all causes, one for death of somatic causes (death of other causes treated as censored observations), and one for death of other causes where somatic deaths were treated as censored. All covariates in these primary models were based on record information available for all 195 participants in the follow-up study. As data on drug use duration and polydrug use were based on interview information from only about half of the deceased, these variables were not included in the primary model. Instead, they were analysed in separate models including baseline age, sex, and one substance use variable at the time. Except for alcohol, where “years of dependence” was chosen, “years of substance use” was preferred. Cox regression analysis was performed in SPSS v. 26. We used 5% significance level and calculated 95% CIs.

## Results

### Sample Characteristics

#### Demography

Baseline age and age at first OAT entry were higher among the deceased, and there was an excess of male participants among the dead (Table 1). Mean age at death was 49.8 years, 50.8 among somatic deaths, and 46.3 among deaths of other causes. Except age at death, the demographic characteristics did not differ significantly between those who died of somatic diseases and other causes (online suppl. Table [ST] 1; for all online suppl. material, see www.karger.com/doi/10.1159/000525694).

### OAT Characteristics

Median time on OAT was 43% lower among the dead. History of OAT interruption did not differ between the groups (Table 1). Among the dead, a smaller fraction (although statistically non-significant) of those who died of somatic disease had experienced OAT interruption compared with the rest, 23% versus 47% (online suppl. Table [ST] 1). A significantly greater fraction of those who started OAT early (1998–2002) compared to late (2003–2007) died (Table 1). The mean age at first OAT entry was significantly higher among the early starters (38.4 years, SD 6.4 years) compared to the late starters (35.1 years, SD 6.7 years, Independent-Samples *t* test, *p* = 0.001, not presented in table).

### Retention in Treatment

Among the 195 participants in the follow-up study, median time in the study was 12.7 years. Of the total 2,442 PY, 2,109 (86.4%) were on and 221 (9.0%) off OAT, while OAT status was unknown for 112 PY (4.6%). Thus, 90.5% of the time with known OAT status was on OAT. One-hundred-and-seventy-five patients had no periods of unknown OAT status and 112 of them (64%) had no history of OAT interruption and a median time on OAT of 12.3 years.

### Health Characteristics

There were no significant differences in the rate of somatic hospital treatment episodes between the dead and alive or between those who died of somatic causes versus other causes. The rate of in-patient psychiatric treatment episodes was significantly higher among those alive; mean rate was 12.2 episodes per 100 PY among those alive and 6.2 among the dead (medians are presented in Table 1). The rate was significantly lower for those with somatic cause of death compared to those dead of other causes (means were 3.7 and 9.1, respectively, medians are presented in online suppl. Table [ST] 1).

### Substance Use History

The number of years of alcohol dependence and use of nicotine, amphetamine, heroin and cannabis, and non-medical injecting was significantly higher among the dead compared to those alive. The number of years with benzodiazepine/z-hypnotic use was also higher among the dead, but the difference was non-significant (Table 1). Comparing the same drug use variables between those who died of somatic causes versus other causes, duration of psychoactive substance use was generally higher among those dead of somatic causes, but the differences except for cannabis were non-significant (online suppl. Table [ST] 1). Polydrug use severity was significantly higher among the dead compared to those alive (Table 1) and higher, though not significant, among those who died of somatic causes versus other causes (online suppl. Table [ST] 1).

### CMRs and SMRs

CMRs per 100 PY by cause of death for the total observation period were somatic cause 0.88 (0.56–1.31), drug-induced cause 0.44 (0.23–0.76), and traumatic cause 0.24 (0.10–0.50), and the all-cause CMR was 1.64 (1.19–2.20) (Table 2). The off-versus-on-OAT all-cause mortality ratio was 2.31 (1.00–4.85) (Table 2). For the whole observation period, all-cause SMR adjusted for age and sex was 8.4 and almost equal for men and women adjusted for age (Table 2).

### Causes of Death

Twenty-two of the 41 deaths (54%) were due to somatic disease, 11 (27%) were drug-induced, and six died of traumatic causes (15%). On OAT, 61% of deaths with known cause were due to somatic disease, 23% to drug-induced causes, and 16% to traumatic causes compared to 38%, 50%, and 13%, respectively, off OAT. Two sudden deaths were categorized as unknown cause, both on OAT. Twelve of the 22 deaths of somatic cause were due to cancer and liver disease, the rest to cardiovascular and respiratory diseases and bacterial infections (Table 3).

### Factors Associated with Mortality Risk

Higher baseline age and rate of somatic hospital treatment episodes were independently associated with *increased* all-cause mortality risk, while increasing rate of in-patient psychiatric treatment episodes was associated with *reduced* risk. When those who died of somatic causes were compared with the rest, higher baseline age was associated with *increased* mortality risk. When comparing those who died of other causes with the rest, increasing rate of somatic hospital treatment episodes was associated with *increased* mortality risk (Table 4).

Associations between all-cause mortality risk and years of alcohol dependence and years of use of nicotine, amphetamines, cannabis, heroin, and drug injecting, respectively, as well as severity of polydrug use, were statistically significant in bivariate analysis, while use of benzodiazepines/z-hypnotics was not (online suppl. Table [ST] 2). The association between *increased* mortality risk and years of alcohol dependence and nicotine and cannabis use and severity of polydrug use remained significant when adjusted for baseline age and sex (online suppl. Table [ST] 2).

## Discussion

### Main Findings

The median observation period was 12.7 years. All-cause CMR was 1.64 per 100 PY for the total observation period. Retention in treatment was high, and the all-cause CMR off OAT was 2.3 times higher than on OAT. This is primarily explained by the reduction in overdose deaths while on OAT. SMR for the total observation period was 8.3 for both men and women adjusted for age. In adjusted analysis, rate of hospital treatment episodes for somatic diseases and baseline age was independently associated with increased all-cause mortality risk, while increasing rate of in-patient psychiatric treatment episodes was associated with reduced risk.

### Mortality Rates

The all-cause CMR on OAT was higher, while the off-versus-on-OAT rate ratio in our study was in line with recent meta-analyses [2, 3]. This likely reflects the high mean age in the cohort and the increasing mortality especially of somatic causes, among ageing OAT patients.

### Mortality and Retention in Treatment

Our results are consistent with the well-established finding that all-cause CMR is substantially reduced on compared to off OAT, largely due to reduction in fatal overdoses [2] but also to reduced traumatic and somatic mortality [5]. Although some patients succeed to taper agonist medication and continue life without harmful use of opioids and other drugs, the mortality-reducing effect of OAT is principally an on-treatment effect. Hence, to assess the overall effect of OAT on mortality, it is essential to evaluate the extent to which OAT programmes retain patients on agonist treatment. Further, mortality must be examined from an intention-to-treat perspective, not only on and off treatment. In this cohort, the retention in treatment was very high over a long period of time. The difference between all-cause CMR on OAT and during the whole on + off-OAT period was only 11%, unlike 106% between the on + off-OAT and off-OAT periods, illustrating the importance of retaining patients in treatment.

### Psychiatric Morbidity and Mortality Risk

It is well-established that concurrent SUDs are associated with more than doubled SMRs among patients with severe psychiatric disorders like bipolar disorder [24] and schizophrenia [22, 23]. The impact of psychiatric comorbidity on mortality among opioid-dependent people and OAT patients is less studied. A study of drug-related mortality on and off methadone treatment from Scotland found that a history of psychiatric admission was strongly associated with increased risk of drug-related death (mainly overdoses) both on and off OAT [27]. A study of mortality among all OAT patients in Sweden 2005–2012 (21,438 PY, 68% on and 32% off OAT), showed − regarding the whole observation period − a significant association between history of in-patient psychiatric treatment and increased non-overdose mortality (comprising deaths of somatic, traumatic, and unknown causes), but not with overdose and all-cause mortality [28]. Our study showed a significant association between increasing rate of psychiatric admissions and reduced all-cause mortality and also a non-significant association with reduced somatic as well as non-somatic mortality. These findings may seem counterintuitive and should be interpreted with caution. *First*, we have measured psychiatric admissions and not the burden of psychiatric morbidity as such. Rate of psychiatric admissions may not be a good proxy for severe psychiatric morbidity, not least among people with problematic substance use. Mental disorders may be overshadowed by SUD-related deviant behaviour and thus remain unrecognized and untreated, leading to underestimation of the impact of psychiatric morbidity on mortality in opioid-dependent populations. *Second*, patients receiving specialist treatment for psychiatric disorders probably get closer follow-up, which may reduce lifestyle factors harmful to health and also promote treatment of chronic diseases and thus reduce mortality. *Third*, some psychiatric disorders may lead to avoidant behaviour that, on the one hand, may reduce high-risk activities associated with increased mortality risk but, on the other hand, lead to less treatment-seeking for mental problems.

If we assume that a substantial part of the psychiatric burden in this population is unrecognized and untreated, our finding may imply that psychiatric treatment leads to reduced mortality. The impact of mental illness and psychiatric treatment on mortality should be further studied.

### Clinical Implications

To minimize mortality, it is essential to keep OAT patients in treatment. Unstable and drug-taking patients should be stabilized and not discharged. OAT providers should not encourage patients to exit OAT. However, when stable patients decide to taper and finally leave OAT, they should have proper long-time follow-up and an open return to OAT.

As OAT reduces particularly fatal overdose but also other causes of death, people on OAT are more likely to reach higher age than their peers not on OAT. This increases the importance of somatic disease, which is a major cause of death in ageing OAT populations [29]. Hence, efforts to prevent and to strengthen early diagnosis, treatment, and follow-up of chronic somatic disorders are important to reduce premature deaths and should be an important task for general practitioners and OAT providers treating ageing OAT patients.

Duration of psychoactive substance use is associated with increased risk of death by somatic causes. Especially nicotine use, mainly cigarette smoking, plays a major role. Efforts to help patients stop smoking and harmful alcohol and drug use are important to reduce lifestyle-related somatic mortality.

### Strengths and Limitations

#### Strengths

The strengths of the study are high baseline participation, long observation period, high follow-up rate, and rich data sources. The high retention in OAT made it possible to investigate long-term mortality during OAT.

#### Limitations

A relatively low number of participants limits the statistical power. This brings about a risk for underestimation of mortality predictors that might have been statistically significant in a bigger sample (type II errors), e.g., duration of use of other substances than nicotine, alcohol, and cannabis. Although this sample is regarded as representative of Norwegian OAT patients, the catchment area comprises no bigger cities, which may influence the external validity of the study. The very high retention rate may also affect the generalizability to OAT programmes with lower retention.

Interview data and data about somatic treatment episodes run until 2007–2009, while data on mortality and psychiatric/OAT treatment run until 2016. However, as both interview and somatic treatment data comprise before-and-on-OAT periods for all participants, the impact of the different time periods is limited and probably without decisive influence on the mortality risk analysis.

Accurate data about switching between OAT medications were lacking. Time on methadone versus buprenorphine could therefore not be included as a covariate.

## Conclusion

In this cohort of ageing OAT patients and with high retention in OAT, we found a 57% mortality rate reduction on compared to off OAT and a majority of deaths due to somatic causes on OAT. Retaining patients in treatment, while enhancing early diagnosis, treatment, and follow-up of chronic somatic diseases and encouraging health-promoting lifestyle changes will further improve outcomes, not least among elderly patients.

## Statement of Ethics

The study was approved by the Regional Committee for Medical and Health Research Ethics, South East Norway, Oslo, Norway (ID 2014/1936 C). All participants alive gave their written informed consent before taking part in the original study and could decline participation in the follow-up. Exemption from professional secrecy duty of confidentiality for the dead was given by the Norwegian Directorate of Health (original study) and the Ethics Committee (above, follow-up study).

## Conflict of Interest Statement

Dr. Helge Waal is an Editorial Board Member of European Addiction Research. The other authors declare no conflicts of interest.

## Funding Sources

The study was funded by the Innlandet Hospital Trust, Brumunddal, Norway (Grants No. 150306/150924).

## Author Contributions

Study conception and design: Ivar Skeie, Thomas Clausen, Arne Jan Hjemsæter, Bent Monsbakken, Anne Signe Landheim, Magne Thoresen, and Helge Waal. Acquisition of data: Ivar Skeie and Bent Monsbakken. Analysis and interpretation of data: Ivar Skeie and Magne Thoresen. Drafting of the manuscript: Ivar Skeie. Critical revision: all authors.

## Data Availability Statement

Data cannot be shared for confidentiality reasons. Queries about the data should be directed to the corresponding author.

## Supplementary Material

Supplementary data

## Figures and Tables

**Table 1:** Sample characteristics by dead/alive at end of study

	*N*	All	Alive	Dead	*p* value
Cohort	195		154	41	

*Demographics*					
Age at start of study (1 January 1998)	195	31.7 (7.6)	30.5 (7.5)	36.1 (6.3)	<0.001^1^, *
Age at OAT start	195	36.8 (6.8)	35.9 (6.7)	40.1 (6.0)	<0.001^1^, *
Age at death				48.5 (6.8)	
Sex/male	195	130 (66.7)	97 (63.0)	33 (80.5)	0.035^2^*
1 year or more ordinary employment^3^	128				
No		30 (24.4)	28 (26.2)	2 (9.5)	0.100^2^
Yes		98 (76.6)	79 (73.8)	19 (90.5)	
Completed 7–9 years of compulsory schooling^3^	135				
No		22 (16.3)	18 (15.9)	4 (18.2)	
Yes		113 (83.7)	95 (84.1)	18 (81.8)	0.794^2^
Completed 12 years of high school^3^	135				
No		91 (67.4)	78 (69.0)	13 (59.1)	
Yes		44 (32.6)	35 (31.0)	9 (40.9)	0.363^2^

*OAT characteristics*					
Years in study^4^	195	12.7 (1.6–17.8)	13.2 (9.0–17.8)	7.9 (1.6–14.2)	<0.001^5^, *
Years on OAT	195	11.3 (1.6–17.8)	12.0 (2.3–17.8)	6.8 (1.6–14.3)	<0.001^5^, *
Experienced OAT interruption 1998–2016	195				
No		126 (64.6)	98 (63.6)	28 (68.3)	
Yes		69 (35.4)	56 (36.4)	13 (31.7)	0.650^2^
First OAT start − period	195				
1998–2002		98 (50.3)	69 (44.8)	29 (70.7)	
2003–2007		97 (49.7)	85 (55.2)	12 (29.3)	0.003^2^, *

*Health characteristics*					
Somatic hospital treatment episodes before and during OAT, rate of episodes per 100 PY^6^	194	40.0 (0–280)	40.0 (0–280)	50.0 (0–220)	0.216^5^
In-patient psychiatric treatment episodes 2000–2016, rate of episodes per 100 PY	191	5.9 (0–130.1)	5.9 (0–130.1)	0 (0–59.7)	0.042^5^, *

*Years of substance dependence (alcohol) or non-medical use (other substances) until 2007–2008* ^3^			
Smoking nicotine	128	28.4 (9.0)	27.1 (8.3)	34.9 (9.5)	0.002^1^, *
Alcohol dependence	127	0 (0–36)	0 (0–36)	7 (0–30)	0.000^5^, *
Benzodiazepine/*z*-hypnotic use	123	18.8 (9.8)	18.3 (9.6)	21.8 (11.0)	0.156^1^,
Amphetamine use	128	12.5 (0–36)	11.0 (0–33)	19.0 (0–36)	0.021^5^, *
Cannabis use	128	19.0 (0–45)	19.0 (0–45)	28.0 (0–40)	0.001^5^, *
Heroin use	127	12.2 (SD 6.7)	14.0 (0–35)	18.9 (6–35)	0.011^5^, *
Injecting drugs	128	16.2 (SD 7.9)	15.1 (SD 7.6)	21.6 (SD 7.3)	0.001^1^, *
Polydrug use^7^	117	4 (1–6)	4 (1–5)	4 (2–6)	0.010^5^, *

Number (%) in categorical variables, mean (standard deviation − SD) in normally distributed^8^, and median (min-max) in non-normally distributed continuous variables. *N* = 195, follow-up cohort^9^.

* Statistically significant difference, *p* < 0.05.

1 Independent-Samples *t* test.

2 Pearson χ^2^ test.

3 Interview information 2007–2008.

4 Total time in study: 2,109 patient years (PY) on, 221 PY off, and 112 PY with unknown OAT status.

5 Mann-Whitney U test.

6 In- and out-patient acute/subacute somatic hospital treatment episodes as of the last 5 years prior to the first OAT entry and up to the five first years on OAT in one or consecutive periods.

7 Number of substances with more than 5 years of dependence (alcohol) or non-medical use (opioids, amphetamines, benzodiazepines, cocaine, or cannabis) until 2008, score from 0 to 6.

^8^Kolmogorov-Smirnov normality test.

^9^The original 2008 cohort comprised 200 participants who had started OMT 1998–2007, 5 persons declined to participate in the 2016 follow-up study.

**Table:** (a) CMRs for the total observation period by death cause (2,508 PY,*N*= 200 [cohort in the original study 1998–2008/09^1^])

			CMR	95% CI
Somatic cause			0.88	(0.56–1.31)
Drug-induced deaths^2^			0.44	(0.23–0.76)
Traumatic causes (suicide, unintended accidents, homicide)			0.24	(0.10–0.50)
All-cause^3^			1.64	(1.19–2.20)

**Table:** (b) All-cause CMR in the on-OAT, off-OAT, and on + off-OAT periods and off-versus-on mortality rate ratio (^4^, ^5^ observation period: on + off OAT 2330 PY, on OAT 2109 PY, and off OAT 221 PY; 112 PY with unknown OAT status were not included; ^6^*N* = 195 [cohort in the 2016 follow-up study])^1^

CMR, period with known OAT status (on + off OAT)	CMR on OAT	CMR off OAT	Off/on OAT rate ratio	*p* value
1.76 (1.28–2.36)	1.57 (1.10–2.17)	3.62 (1.68–6.87)	2.31 (1.00–4.85)	0.050^7^

**Table:** (c) All-cause SMRs by sex and age groups (^4^, ^8^ total observation period = 2508 PY, *N* = 200 [cohort in original study 1998–2008/09])

Age group		Total	Male	Female
20–44 years		7.7 (3.9–13.7)	8.0 (3.7–15.2)	6.7 (1.1–22.0)
45–69 years		5.0 (3.4–7.0)	5.0 (3.3–7.3)	4.7 (1.9–9.8)
20–69 years		8.4 (6.1–11.2)	8.4 (5.8–11.6)	8.3 (3.9–15.8)

1 The original cohort comprised 200 participants who had started OMT as of 1 January 1998–30 June 2007 (13 had died before the cohort was established in 2007–2008), 5 persons declined to participate in the 2016 follow-up study.

2 Following the definition of the European Monitoring Centre for Drugs and Drug Addiction (EMCDDA), mostly fatal overdoses.

3 Two deaths of unknown cause included in all-cause CMR.

4 OpenEpi (http://www.openepi.com), accessed 30 March 2020.

5 Off-versus-on mortality rate ratio for cause of death categories is not presented due to low number of deaths.

6 No deaths occurred in the period with unknown OAT status.

7 Mid *P* exact test.

8 SMRs for death cause categories and on and off OAT are not presented due to low number of deaths.

**Table 3:** Causes of death on and off OAT

	All deaths	On OAT	Off
Cardiovascular	3	3	0
Cancer − not liver	7	6	1
Liver including liver cancer	5	4	1
Bacterial infections	3	2	1
Resp. disease	4	4	0
Somatic causes − total	**22 (53.7)**	**19**	**3**
Drug-induced cause (overdoses/SUD)	**11 (26.8)**	**7**	**4**
Suicide	2	1	1
Unintentional accidents	1	1	0
Homicide	3	3	0
Traumatic causes − total	**6 (14.6)**	**5**	**1**
Unknown cause1	**2 (4.9)**	**2**	**0**
All	**41 (100)**	**33**	**8**

Values are numbers with percentages in parentheses. 2,109 patients-years (PY) on OAT, 221 PY off OAT, 112 PY unknown OAT status^2^. *N* = 195 (cohort in the 2016 follow-up study^3^).

^1^Both deaths were sudden deaths without certain cause.

^2^No deaths occurred in the period with unknown OAT status.

^3^The original cohort comprised 200 participants who had started OMT as of 1 January 1998 until 30 June 2007 (13 had died before the cohort was established in 2008), 5 persons declined to participate in the 2016 follow-up study.

**Table 4:** Factors associated with risk of death after the first entry to OAT

	HR (95% CI)	*p* value	aHR (95% CI)	*p* value
**(a) All-cause mortality**				
Age at start of study (1 January 1998)	1.08 (1.04–1.13)	0.000*	1.07 (1.02–1.13)	0.004*
Sex				
Women	1			
Men	2.27 (1.05–4.90)	0.038*	1.85 (0.84–4.07)	0.126
First OAT start − period				
2003–2007	1			
1998–2002	1.91 (0.96–3.80)	0.066	1.15 (0.53–2.48)	0.724
In-patient psychiatric treatment episodes 2000–2016, rate of episodes per 100 PY	0.98 (0.95–1.01)	0.114	0.97 (0.95–1.00)	0.037*
Somatic hospital treatment episodes before and during OAT,^1^ rate of episodes per 100 PY	1.01 (1.00–1.01)	0.087	1.01 (1.00–1.01)	0.005*

**(b) Death of somatic cause^2^**				
Age at start of study (1 January 1998)	1.11 (1.04–1.17)	0.001*	1.12 (1.04–1.20)	0.001*
Sex				
Women	1			
Men	3.45 (1.02–11.65)	0.047*	2.37 (0.68–8.27)	0.175
First OAT start − period				
2003–2007	1			
1998–2002	1.21 (0.48–3.03)	0.683	0.55 (0.20–1.49)	0.240
In-patient psychiatric treatment episodes 2000–2016, rate of episodes per 100 PY	0.95 (0.89–1.01)	0.084	0.95 (0.89–1.00)	0.061
Somatic hospital treatment episodes before and during OAT,^1^ rate of episodes per 100 PY	1.00 (0.99–1.01)	0.558	1.01 (1.00–1.02)	0.076

**(c) Deaths of overdose, traumatic causes (accidents, suicide, and homicide), and unknown causes^2^**			
Age at start of study (1 January 1998)	1.05 (0.99–1.12)	0.091	1.02 (0.95–1.10)	0.548
Sex				
Women	1			
Men	1.55 (0.56–4.31)	0.400	1.44 (0.51–4.05)	0.495
First OAT start − period				
2003–2007	1			
1998–2002	3.30 (1.09–10.00)	0.035	2.86 (0.83–9.80)	0.095
In-patient psychiatric treatment episodes 2000–2016, rate of episodes per 100 PY	0.99 (0.97–1.02)	0.618	0.98 (0.96–1.01)	0.271
Somatic hospital treatment episodes before and during OAT,^1^ rate of episodes per 100 PY	1.01 (1.00–1.02)	0.062	1.01 (1.00–1.02)	0.029*

Unadjusted hazard ratio (HR) and adjusted hazard ratio (aHR) for death during the whole observation period. 2,109 patient-years (PY) on, 221 PY off, and 112 PY with unknown OAT status. Cox regression model. *N* = 195 (cohort in the 2016 follow-up study).

* Statistically significant difference, *p* < 0.05.

1 In- and out-patient acute/subacute somatic hospital treatment episodes as of the last 5 years prior to the first OAT entry and up to the five first years on OAT in one or consecutive periods.

2 Participants who had died of somatic and other causes, respectively, were compared with the rest of the cohort.

## Notes

Present address: Arne Jan Hjemsæter, Psykologspesialist Arne Jan Hjemsæter, Hamar, Norway.
