# Supplementary material for: Mortality, Causes of Death, and Predictors of Death among Patients On and Off Opioid Agonist Treatment: Results from a 19-Year Cohort Study
Source: Eur Addict Res. 2022 Aug 23;28(5):358–67. doi: 10.1159/000525694 (PMC9533433; doi:10.1159/000525694)
Supplement: Supplementary file 1 — Supplementary data [file ear-0028-0358-s01.docx]

**Supplementary table 1**: Sample characteristics by cause of death. Number (%) in categorical variables, mean (standard deviation – SD) in normally distributed^1^ and median (min-max) in non-normally distributed continuous variables. N=41.

Cause of death

Somatic Other

Number of deaths 22 19

*Demographics*

Age at start of study (1 January 1998) 37.4 (4.9) 34.5 (7.5) p=0.146 ^a^

Age at OAT start 41.7 (5.3) 38.4 (6.5) p=0,081 ^a^

Age at death 50.5 (5.4) 46.3 (7.7) p=0.047 ^a *^

Sex/male 19 (86.4) 14 (73.7) p=0,307 ^c^

1 year or more ordinary

employment^2^ 12 (92.3) 7 (87.5) p=0.716 ^c^

Completed 7-9 years

compulsory schooling^2^ 12 (85.7) 6 (75.0) p=0.531 ^c^

Completed high school – 12 years^2^ 6 (42.9) 3 (37.5) p=0.806 ^c^

*OAT-characteristics*

Years in study, median (SD) 8.8 (3.5) 7.9 (3.6) p=0.418 ^a^

Years on OAT, median (SD) 8.0 (3.6) 7.4 (3.8) p=0.619 ^a^

Experienced interruption

of OAT (median (SD)

- No 17 (77.3) 10 (52.6)

- Yes 5 (22.7) 9 (47.4) p=0.097 ^c^

First OAT-start – period

1998–2002 14 (63,6) 15 (78,9)

2003-2007 8 (36,4) 4 (21,1) p=0,283^c^

*Health characteristics*

Somatic hospital treatment

episodes before and during OAT^3^,

rate of episodes per 100 PY 35.8 (10.0–193.1) 60.0 (0–220.0) p=0.647 ^b^

In-patient psychiatric treatment

episodes 2000–2016,

rate of episodes per 100 PY 0 (0–59.7) 8.1 (0–58.3) p=0.006 ^b^*

*Years of substance dependence (alcohol) or non-medical use (other substances) until 2007–2008^2^*

Smoking nicotine 35.5 (29–48) 35.0 (2–42) p=0.389 ^b^

Alcohol dependence 11.3 (10.6) 4.3 (4.6) p=0.115 ^a^

Benzodiazepine/z-hypnotic use 23.9 (9.4) 18.1 (13.3) p=0.283 ^a^

Amphetamine use (median) 20.3 (9.8) 13.3 (9.5) p=0.135 ^a^

Cannabis use (median) 30.6 (6.9) 17.3 (14.4) p=0.009 ^a *^

Heroin use (mean) 20.3 (11–35) 16.1 (6–27) p=0.349 ^b^

Injecting drugs (mean) 22.6 (SD 7.0) 19.6 (8.0) p=0.388 ^a^

Polydrug use^4)^ 5 (4–6) 4 (2–5) p=0.078 ^b^

* Statistically significant difference, p<0.05

^a^) Independent samples T-test

^b^) Mann-Whitney U test

^c^) Pearson Chi-Square

^1^ Kolmogorov-Smirnov normality test

^2^ Interview information 2008 from 123–128 (different items) participants comprising 19–21 of totally 41 dead

^3^ In- and out-patient acute/subacute somatic hospital treatment episodes as of the last five years prior to the first OAT entry and up to the five first years on OAT in one or consecutive periods.

^4^ Number of substances with more than five years of dependence (alcohol) or non-medical use (opioids, amphetamines, benzodiazepines, cocaine or cannabis) until 2008, score from 0 to 6.

**Supplementary Table 2:** Associations between mortality risk and years of substance dependence (alcohol) or non-medical substance use until 2008 adjusted for age at start of study (1 January 1998) and sex (male). Unadjusted hazard ratio (HR) and adjusted hazard ratio (aHR) for death during the whole observation period. N 123–128, interview information^1^.

Number Number HR (95% CI) aHR (95% CI)

interviewed dead

Smoking nicotine 128 21 1,10 (1.04­–1.16) p=0.001** 1.23 (1.08–1.40) p=0.002*

Age 1.08 (1.04–1.13) p=0.000 0.87 (0.77–1.00) p=0.042

Sex 2.26 (1.05–4.90) p=0.038 1.72 (0.58–5.15) p=0.331

Alcohol dependence 127 21 1.08 (1.04–1.12) p=0.000*** 1.07 (1.02–1.11) p=0.004*

Age 1.08 (1.04–1.13) p=0.000 1.05 (0.98–1.12) p=0.161

Sex 2.26 (1.05–4.90) p=0.038 1.40 (0.44–4.45) p=0.570

Benzodiazepine/z-hypnotic use 123 19 1.03 (0.98–1.08) p=0.243 1.01 (0.96–1.05) p=0.742

Age 1.08 (1.04–1.13) p=0.000 1.07 (1.00–1.14) p=0.056

Sex 2.26 (1.05–4.90) p=0.038 1.56 (0.51–4..81)p=0.436

Amphetamine use 128 21 1,05 (1.01–1.10) p=0.025* 1.03 (0.98–1.08) p=0.218

Age 1.08 (1.04–1.13) p=0.000 1.06 (0.99–1.13) p=0.089

Sex 2.26 (1.05–4.90) p=0.038 1.50 (0.48–4.71) p=0.487

Cannabis use 128 21 1.08 (1.03–1.13) p=0.001** 1.06 (1.00–1.12) p=0.034*

Age 1.08 (1.04–1.13) p=0.000 1.04 (0.97–1.11) p=0.279

Sex 2.26 (1.05–4.90) p=0.038 1.18 (0.36–3.82) p=0.785

Heroin use 127 21 1.08 (1.02–1.15) p=0.012* 1.05 (0.98–1.12) p=0.160

Age 1.08 (1.04–1.13) p=0.000 1.05 (0.98–1.12) p=0.175

Sex 2.26 (1.05–4.90) p=0.038 1.64 (0.54–5.05) p=0.385

Injecting drugs 128 21 1.09 (1.03–1.16) p=0.003* 1.06 (0.99–1.15) p=0.112

Age 1.08 (1.04–1.13) p=0.000 1.03 (0.95–1.11) p=0.459

Sex 2.26 (1.05–4.90) p=0.038 1.52 (0.49–4.73) p=0.473

Polydrug use^2)^ 117 19 2,21 (1,20–4,08) p=0,011* 2,10 (1,13–3,90) p=0.019*

Age 1.08 (1.04–1.13) p=0.000 1,08 (1,01–1,15) p=0.022

Sex 2.26 (1.05–4.90) p=0.038 0.98 (0.30–3.18) p=0.975

^1^) Interview information 2008 from 123–128 (different items) participants comprising 19–21 of totally 41 dead

^2^) Number of substances with more than five years of dependence (alcohol) or non-medical use (opioids, amphetamines, benzodiazepines, cocaine, or cannabis) until 2008, score from 0 to 6.

* HR, drug use variable statistically significant

Comment: Cox regression models for risk of somatic death cause versus the rest of the cohort (alive at end of study + dead of causes other than somatic disease) show significantly increased HR for years of cannabis use, years of alcohol dependence, years of nicotine smoking and severity of polydrug use adjusted for age and sex. For death of other causes than somatic disease versus the rest of the cohort there is no significant impact on HR for any of the drug use variables, adjusted for age and sex. Tables available from the first author.
